# Forecasting the Effects of Land Use Scenarios on Farmland Birds Reveal a Potential Mitigation of Climate Change Impacts

**DOI:** 10.1371/journal.pone.0117850

**Published:** 2015-02-20

**Authors:** Karine Princé, Romain Lorrillière, Morgane Barbet-Massin, François Léger, Frédéric Jiguet

**Affiliations:** 1 Muséum National d’Histoire Naturelle, UMR 7204 MNHN-CNRS-UPMC, Centre de Recherches sur la Biologie des Populations d’Oiseaux, CP 51, Paris, France; 2 Department of Forest and Wildlife Ecology, University of Wisconsin-Madison, Madison, Wisconsin, United States of America; 3 Department of Ecology and Evolutionary Biology, Yale University, New Haven, Connecticut, United States of America; 4 AgroParisTech, UMR SAD-APT INRA/AgroParisTech, Paris, France; University of Colorado, UNITED STATES

## Abstract

Climate and land use changes are key drivers of current biodiversity trends, but interactions between these drivers are poorly modeled, even though they could amplify or mitigate negative impacts of climate change. Here, we attempt to predict the impacts of different agricultural change scenarios on common breeding birds within farmland included in the potential future climatic suitable areas for these species. We used the Special Report on Emissions Scenarios (SRES) to integrate likely changes in species climatic suitability, based on species distribution models, and changes in area of farmland, based on the IMAGE model, inside future climatic suitable areas. We also developed six farmland cover scenarios, based on expert opinion, which cover a wide spectrum of potential changes in livestock farming and cropping patterns by 2050. We ran generalized linear mixed models to calibrate the effects of farmland cover and climate change on bird specific abundance within 386 small agricultural regions. We used model outputs to predict potential changes in bird populations on the basis of predicted changes in regional farmland cover, in area of farmland and in species climatic suitability. We then examined the species sensitivity according to their habitat requirements. A scenario based on extensification of agricultural systems (i.e., low-intensity agriculture) showed the greatest potential to reduce reverse current declines in breeding birds. To meet ecological requirements of a larger number of species, agricultural policies accounting for regional disparities and landscape structure appear more efficient than global policies uniformly implemented at national scale. Interestingly, we also found evidence that farmland cover changes can mitigate the negative effect of climate change. Here, we confirm that there is a potential for countering negative effects of climate change by adaptive management of landscape. We argue that such studies will help inform sustainable agricultural policies for the future.

## Introduction

The modification and management of landscapes to produce food or other agricultural commodities for human consumption represents one of the most severe and widespread threats to global biodiversity [1]. In 2011 in the European Union, 34% of the terrestrial area was used for cropland and 14% for grassland [2]. Numerous studies have highlighted that during the last decades agricultural intensification has already dramatically affected water quality, wildlife habitats and biodiversity [3–6], and this is particularly well documented for farmland birds [7–10]. In Europe, declines in farmland birds have been severe, and many species have suffered large decreases in abundance since the 1980s, such as skylark *Alauda arvensis* (-51%), linnet *Carduelis cannabina* (-63%), yellowhammer *Emberiza citrinella* (-44%) and whinchat *Saxicola rubetra* (-71%) [11].

To respond to increases in major human needs over the next decades (changes in global trade, technology, policies), the structure of agricultural production and spatial patterns in farmland are likely to undergo important transformations [12]. Within agricultural areas, the increasing demand for food production, the introduction and expansion of bio-energy crops, modernization of agriculture techniques, abandonment of grazing areas and crop specialization and intensification, are main factors affecting farmland cover. These land cover changes are characterized by several significant processes such as the transformation of agricultural landscapes into new combinations of crops and semi-natural elements or the management of these crops to increase their productivity [4, 13, 14]. Furthermore, the choices in public policies and the spatial scale at which they are implemented (nationally or regionally) can imply differences in spatial patterns of land cover changes. In addition to this, the debate is still open between ‘land sharing’, that aims to integrate goals for food production and biodiversity protection on the same land, and ‘land sparing’, that aims to separate intensive farming from protected ecosystems at the larger scale [e.g. 15, 16]. Thus, in this context, authorities need tools for decision support, to propose public policies to reconcile agricultural production and biodiversity [17].

Anthropogenic climate change is another major driver of current biodiversity changes [18]. Evidence is accumulating that climate change in recent decades [19] has already affected many plants and animals [20–22]. A well-documented effect of climate change is a redistribution of species ranges as they track moving climate envelopes [23–25]. In the future, anthropogenic climatic change is expected to result in warmer global conditions from 2° to 4°C by 2100 [best estimate for RCP2.6 and RCP8.5 scenarios, 26]. The ability of species to survive this climatic transition may depend on the availability of suitable habitats within their future ranges and the ability for species to reach these new areas or adapt [27–29]. The availability of these habitats will also depend on changes in future land use and farmland cover.

Only few studies have explored the potential impacts of combined future climate and land use changes on biodiversity at national and continental scales [but see 28, 30, 31], because of the scarcity of relevant non-climatic data projected in the future [32]. However, non-climatic factors such as land-use intensity or landscape structure may be determinant for species richness [33] and may also affect large scale species distributions [34]. Moreover, Eglington & Pearce-Higgins [35] have recently highlighted that the impacts of land-use change can exceed climate change impacts on some species. Thus, climate and land use changes have to be considered together to adequately predict changes in biodiversity [36]. So far, studies that have forecasted the combined potential impacts of future climate and land use changes have often been based on species distributions models (SDMs), applied to presence/absence distribution data [e.g. 28, 37]. However, population size and associated trends are the most frequently used data to assess the conservation status of species, to develop biodiversity indicators and determine priorities for action [38].

The objective of this study was to assess the potential impacts of predicted future changes in farmland cover, area of farmland habitats and climate for 2050 on bird populations in French farmland. We focused on breeding birds, a group which is widely used as an indicator of environmental changes at the continental scale [39]. We relied on projections from SDMs and from the IMAGE model [40], based on Special Report on Emission Scenarios SRES [41], respectively, to integrate likely changes in species climatic suitability and changes in proportion of farmland habitats included in the geographical projection of the future suitable climatic areas for the corresponding species. Moreover, as mentioned above, land cover scenarios are rare. To this purpose, we built up farmland cover scenarios to further link bird abundances to cropland/grassland proportions. Data of farmland cover, especially crop patterns, must be analyzed at finer levels than the national level to account for regional disparities [42]. To do this, we developed four main scenarios of farmland cover changes at a fine spatial scale (i.e. French Small Agricultural Regions, SAR): a *‘status quo’* scenario, a *‘biofuel’* scenario, a *‘livestock extensification’* scenario and an *‘extensification’* scenario, exploring different combinations of agricultural policies and environmental commitments projected for 2050. Two of them were also developed at multiple levels of policy implementation (national or regional). Farmland cover scenarios differed in composition and spatial configuration of croplands and grasslands. Here, scenarios of “extensification” of farmland cover correspond to lowest intensive agriculture, through suitable changes in cropland/grassland to reduce pressure from modern agriculture. We hypothesize that: (1) land cover management could play a role in mitigating climate effect on farmland bird populations [35]; (2) a scenario with a regional level of implementation, leading to spatially heterogeneous policies, could benefit more bird populations than a global scenario with policies uniformly implemented at national scale; (3) depending on the ecological requirements of bird species (habitat specialization), their capacity to respond to climate change will be affected by the coverage of agricultural land and the agricultural policies [43].

## Methods

**Fig 1:**
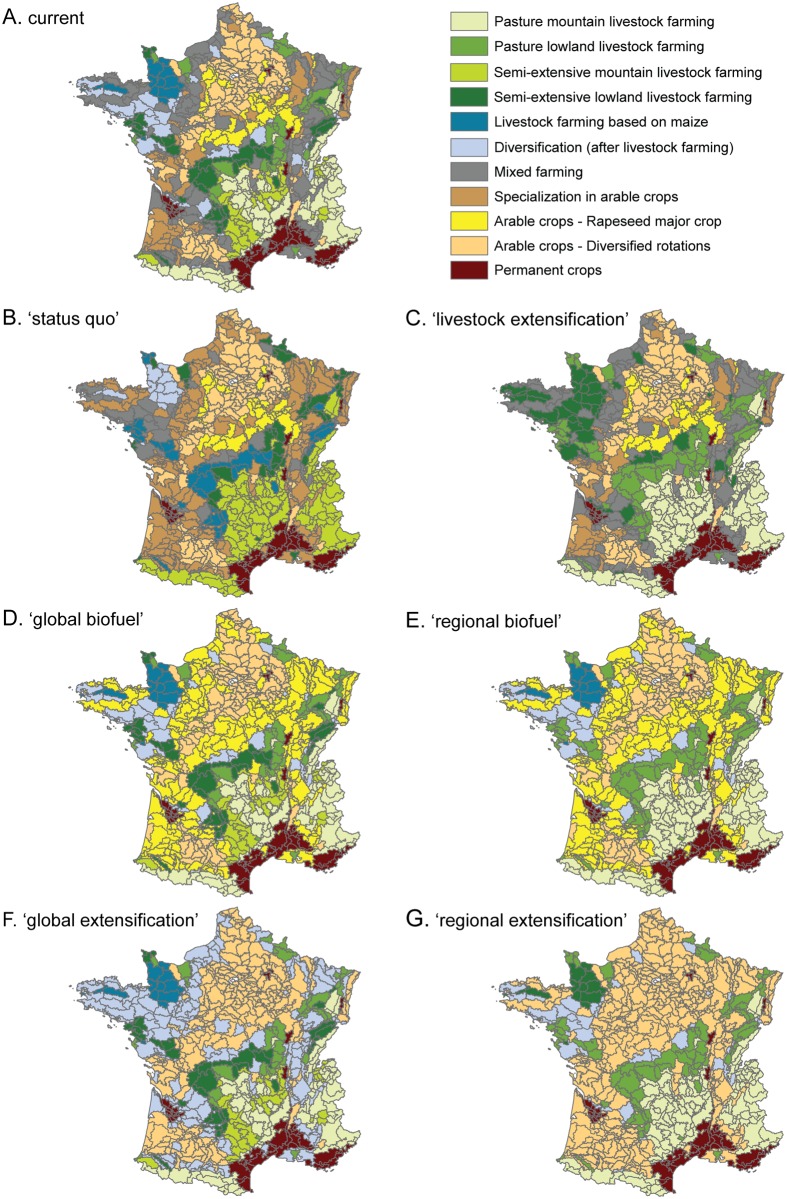
Typology of the main agroecosystems at the scale of small agricultural regions (SAR) in France. (A) Current distribution of main agroecosystems and (B-G) future distribution of main agroecosystems in 2050 according to the six different farmland cover scenarios.

More than half of France is covered by agricultural land, with regional differences in terms of agroecosystems. Croplands and grasslands represent respectively about 66% and 34% of the French agricultural area [44]. We used a typology of the major French agroecosystems (Fig. 1a.), classified based on their structural and dynamic similarities, as reported by AND International [45]. Agroecosystems are defined at the scale of Small Agricultural Regions (SAR), by the main use of the Utilized Agricultural Area (UAA) and the technical and economic orientations of farms. France is divided into 713 SAR according to homogeneous agricultural activities and practices, which range from 11 to 4413 km² (National Institute of Statistics and Economic Studies, INSEE; www.insee.fr). The consistency of these territorial entities at both agro-ecological and economic levels makes them particularly well-suited for our statistical analyses and projected modeling [46].

### Bird abundance data

We used bird data from the French Breeding Bird Survey (BBS), a standardized monitoring program in which skilled volunteer ornithologists identify breeding birds by song or visual contact in spring [47]. Each observer provides the name of a locality, and a 2 x 2 km square to be surveyed is randomly selected within a 10 km radius of this location (i.e. among 80 possible plots). Random selection ensures that surveyed habitats closely match the actual distribution of available habitats in France. In each square, the observer performs 10 point counts, 5 minutes each, separated by at least 300 m twice per spring with 4 to 6 weeks between observation sessions. Counts are repeated annually by the same observer at the same points, at approximately the same date (±7 days from April to mid-June) and the same time of day (±15 minutes). Among the common breeding species monitored by the scheme, we focused on those 34 species (Table 1) classified as farmland specialists and habitat generalists [see 48]. We considered BBS squares that were surveyed at least once between 2001 and 2009 and that included at least 5 of the 10 points located within farmland habitats, according to the habitat noted by the observers in the field [48]. For a given point, the maximum count of the two annual visits is retained as the yearly relative abundance, except for three migrant species meadow pipit *Anthus pratensis*, yellow wagtail *Motacilla flava* and whinchat *Saxicola rubetra*: for these species only counts from the second session were considered given that until early May there are still numerous migrating individuals on the move or late wintering birds (for meadow pipit). The species local relative abundances at the SAR level were obtained by summing the relative abundance of the points in each SAR, each year. The numbers of point counts made per SAR were incorporated into the analyses to account for differences in the regional sampling rate.

**Table 1:** List of the species comprising the bird community examined in this study and their habitat characteristics.

Species	Habitat specialization	SSIg	Main Habitat
Grey Partridge *Perdix perdix*	Farmland	1.25	Cropland
Yellow Wagtail *Motacilla flava*	Farmland	1.33	Cropland
Corn Bunting *Emberiza calandra*	Farmland	1.56	Cropland
Lapwing *Vanellus vanellus*	Farmland	1.56	Cropland
Quail *Coturnix coturnix*	Farmland	1.59	Cropland
Skylark *Alauda arvensis*	Farmland	1.60	Cropland
Red-legged Partridge *Alectoris rufa*	Farmland	1.84	Mixed
Linnet *Carduelis cannabina*	Farmland	1.85	Mixed
Rook *Corvus frugilegus*	Farmland	1.94	Mixed
Meadow Pipit *Anthus pratensis*	Farmland	2.00	Mixed
Whitethroat *Sylvia communis*	Farmland	2.04	Mixed
Kestrel *Falco tinnunculus*	Farmland	2.12	Mixed
Yellowhammer *Emberiza citrinella*	Farmland	2.26	Grassland
Stonechat S*axicola torquatus*	Farmland	2.29	Grassland
Cirl Bunting *Emberiza cirlus*	Farmland	2.37	Grassland
Buzzard *Buteo buteo*	Farmland	2.42	Grassland
Whinchat *Saxicola rubetra*	Farmland	2.44	Grassland
Hoopoe *Upupa epops*	Farmland	2.53	Grassland
Red-backed Shrike *Lanius collurio*	Farmland	2.58	Grassland
Wood Lark *Lullula arborea*	Farmland	2.61	Grassland
Blackbird *Turdus merula*	Generalist		
Blackcap *Sylvia atricapilla*	Generalist		
Blue Tit *Cyanistes caeruleus*	Generalist		
Carrion Crow *Corvus corone*	Generalist		
Chaffinch *Fringilla coelebs*	Generalist		
Cuckoo *Cuculus canorus*	Generalist		
Dunnock *Prunella modularis*	Generalist		
Golden Oriole *Oriolus oriolus*	Generalist		
Great Tit *Parus major*	Generalist		
Green Woodpecker *Picus viridis*	Generalist		
Jay *Garrulus glandarius*	Generalist		
Melodious Warbler *Hippolais polyglotta*	Generalist		
Nightingale *Luscinia megarhynchos*	Generalist		
Wood Pigeon *Columba palumbus*	Generalist		

Habitat specialization (farmland specialist vs. generalist) is given for each species, and specialization index for grasslands (SSIg) and main habitat of specialization are given for each farmland specialist. ‘SSIg’ is the habitat specialization index of each farmland species; the higher it is the more specialized is the species for grasslands. ‘Main habitat’ corresponds to the main habitat of farmland species, determined on the basis of SSIg (see the statistical methods section for more details).

**Fig 2:**
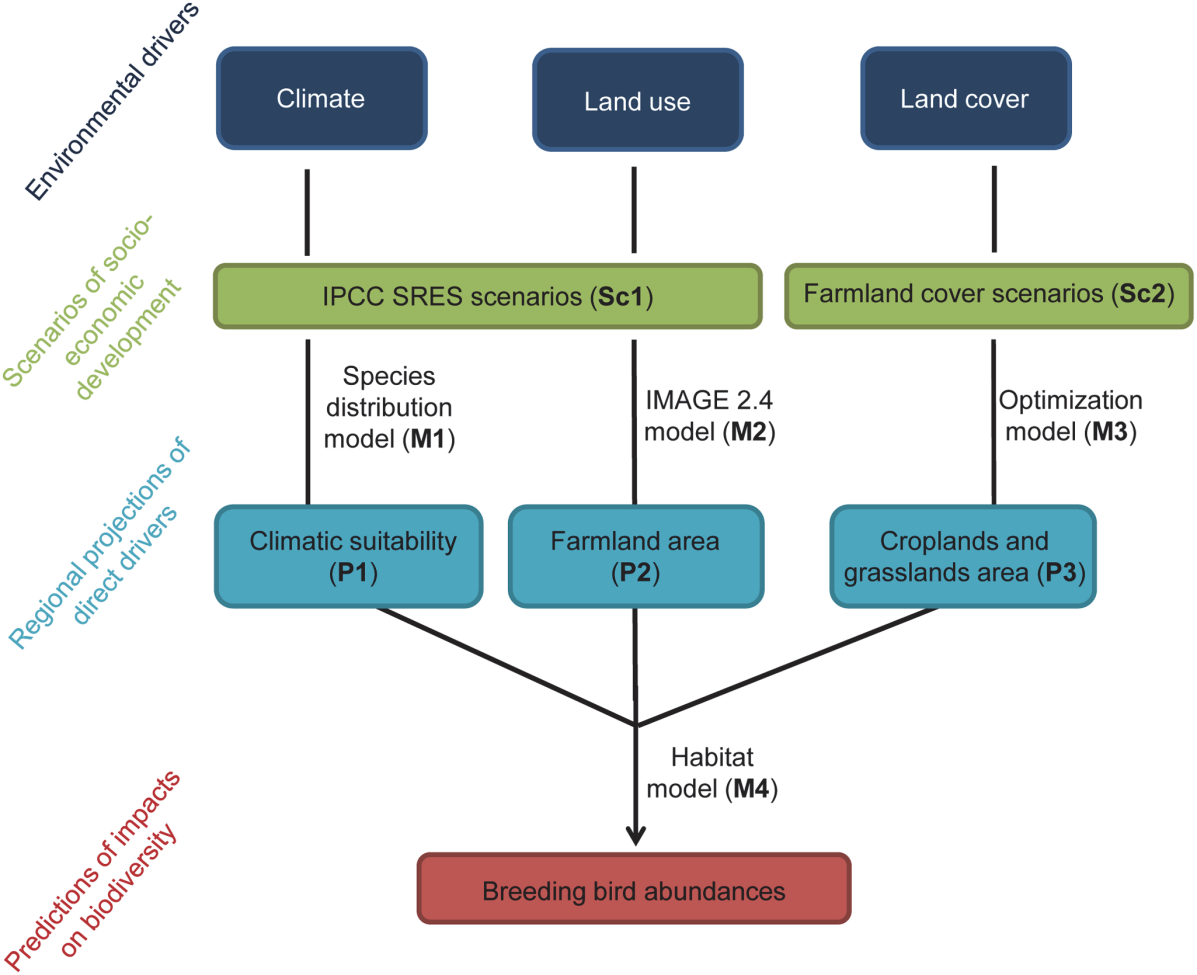
Flowchart summarizing the different steps of the study methods. Overview of the framework to model the effect of climate, land use and land cover changes on farmland bird populations. This flowchart shows how the different environmental drivers, and associated projections (P) derived from various scenarios (Sc), were integrated in our analysis through different methodological tools (M). We used existing scenarios (IPCC-SRES) to derive projections of future climate and land use, while we developed the farmland cover scenarios on the basis of expert judgment to derive projections of future land cover. The regional projections of the three main drivers were then integrated as predictors in a habitat model to forecast changes in breeding bird abundances within French agricultural regions.

Fig. 2 summarizes the different steps of the study methods developed below and how the different environmental drivers, and associated projections, were integrated in our analyses.

### Estimating current and future climatic suitability of each bird species

Values of current (1961–1990 time period) and future (2050) climatic suitability (P1 in Fig. 2) of a species across its whole range were obtained through species distribution modeling techniques (M1 in Fig. 2), relating presence—absence data to climatic variables across the species’ distribution. The presence-absence data-over the Western Palearctic- were obtained by geo-referencing and digitizing maps from the handbooks of the Birds of the Western Palearctic 2006 [49]. The spatial resolution of presence/absence data was 0.5°. Temperature and precipitation are expected to impose direct or indirect constraints on bird distributions [50–52]. So we used the following eight climatic variables in the niche models: (1) annual mean temperature; (2) mean temperature of the warmest month; (3) mean temperature of the coldest month; (4) temperature seasonality; (5) annual precipitation; (6) precipitation of the wettest month; (7) precipitation of the driest month; and (8) precipitation seasonality [28]. The seasonality is the coefficient of variation of the monthly means. These variables were derived from the monthly mean temperatures and precipitation over the intervals 1961–1990 for the current climatic conditions (http://worldclim.org). Future climate projections for 2050 were derived from five general circulation models (BCM2, ECHAM5, HADCM3, MIROHIC3_2-HI, and MK3) and three different emission scenarios (SRES: A1B, B1, and A2, when available; Sc1 in Fig. 2). Because the future predictions were only available at a rough resolution, they were downscaled to the 0.5° resolution (following the method described in http://www.worldclim.org/downscaling). To model species distributions, we used seven different modeling techniques implemented within the BIOMOD package [53] in R: three regression methods (GLM, Generalized Linear Models; GAM, Generalized Additive Models, and MARS, Multivariate Adaptive Regression Splines), a recursive partitioning method (CTA, Classification Tree Analysis), and three machine-learning methods (ANN, Artificial Neural Networks; GBM, Generalized Boosted Models, and RF, Random Forests). In order to evaluate the predictive performance of the SDMs for each species, we used a random subset of 70% of the data to calibrate the model, and then used the remaining 30% for evaluation, using a threshold-independent method, the area under the relative operating characteristic curve (AUC) [54]. The data subsetting approach was replicated five times and was the basis for calculating the mean AUC of the cross-validation (see S1 Table). The projections we used were obtained from models that used 100% of the available data. An ensemble forecast technique was then used to account for variability among distribution modeling techniques and climate models, in order to obtain the central tendency [22, 55]. Current and future consensus distributions were obtained by calculating the weighted mean distributions across SDMs (and the mean across GCMs and SREs): the seven models were ranked according to their predictive performance, and a decay of 1.6 gave the relative importance of the weight, producing respective weights of 0.38, 0.24, 0.15, 0.09, 0.06, 0.04, and 0.02 [56, 57]. The outputs of these models, thereafter referred to as climatic suitability, are estimates of the probability of presence of a species given the climatic conditions at a 0.5° (50 x 50km) spatial resolution. From the modelled distributions, climatic suitability values were first extracted using a GIS (ArcGIS 9.3) by overlapping grid cells and SAR, and then averaged in each SAR weighted by the proportion of SAR included in grid cells. S1 Table presents the average of the current and future climatic suitability values used in this study for each species.

### Estimating the current and future area of farmland habitats

We estimated the proportion of farmland habitats (P2 in Fig. 2) as the percentage of land area within each SAR covered by (1) herbaceous or cultivated pasture, (2) cultivated and managed areas and (3) mosaic cropland/natural vegetation. These land use variables were derived from 19 land cover types available in the IMAGE 2.4 model (M2 in Fig. 2) [40]. The IMAGE 2.4 model is an Earth system model that includes the major feedback mechanisms in the biophysical system. It assumes population and macro-economy as key drivers to establish physical indicators for both the energy/industry system and the agriculture/land-use system for assessment of changes in land cover [40]. This model is developed at a 0.5° resolution grid for all decades since 1960. To be consistent with current climatic variables, we used the average of the variables from 1960 to 1990 to obtain the current proportion of farmland habitats. Although land uses can have changed since this period, we expected the percentage covered by farmland to remain nearly the same, with land use changes at such large spatial scale happening slower than land cover data. Future land cover projections (for 2050) were obtained from the three SRES scenarios, A1B, A2, and B1 of the IMAGE 2.4 model [40]. The future proportion of farmland habitats was obtained by averaging projected proportions from the three SRES scenarios. We downscaled those data at the scale of each SAR (S1 Fig.), similarly to climatic suitability.

We considered a 0.5° resolution (~50x50km scale) for both climate and land use data and associated projections (no scenarios were available for these drivers at a finer spatial scale). However, we considered land cover scenarios and bird abundances at the PRA scale—because they are more tightly linked to local conditions in their realization of abundance. At the scale of the PRA, we considered a homogeneous realization of the scenario, with an average effect on bird populations.

### Farmland cover scenarios

Farmland cover scenarios (Sc2 in Fig. 2) are based on potential realistic land cover changes in the agricultural area within each region (SAR), i.e. changes in livestock farming and cropping patterns between 2000 (the base year for agricultural data) and 2050 (time horizon considered for each scenario). Changes in livestock farming and cropping patterns were here defined as changes in the share (%) of different crop and grassland categories (hereafter broadly referred as crops) within the UAA. Data used to develop farmland cover scenarios came from the national 2000 General Agricultural Census that recorded crop and grassland areas at the municipality scale (www.agreste.agriculture.gouv.fr). Data were aggregated and converted to the proportion of the UAA occupied within each SAR. We considered the following nine crops: permanent grassland, temporary grassland, cereals, grain maize, rapeseed, sunflower, corn fodder, proteaginous (peas, broad beans, lupine, etc.) and forage crops (sorghum, alfalfa, white clover, etc.). In a first step, we defined changes in main agroecosystems within SAR according to each scenario (Fig. 1; see S1 Text for more details). In a second step, we estimated the variations in the proportions of crops within each SAR linked to the changes in the main agroecosystem. For each scenario, we calculated changes in proportions of crops using an optimization method (M3 in Fig. 2) taking various constraints into account (see S1 Text and S3 and S4 Tables). The resulting future crop proportions (P3 in Fig. 2) were then used to predict the impacts of the different scenarios on breeding bird abundances. National changes in crop proportions according to each farmland cover scenarios are given in S5 Table. Management practices (e.g. fertilizer input, yields, etc.) in the different crops were implicitly considered as it is dependent on the land cover scenario we applied. Indeed, as we did not have access to a regional scale product of sufficient resolution across the whole country, we did not integrate these practices in the models, making the assumption that our results are valid if the practices do not change between the current and the future.

We developed four future scenarios, namely *‘status quo’*, *‘biofuel’*, *‘livestock extensification’* and *‘extensification’*. The *‘status quo’* scenario is considered as the reference case. Extensification means a strategy of reducing production costs or inputs leading to a reduction in output for an equivalent surface. The *‘livestock extensification’* and *‘extensification’* scenarios both promote the development of more extensive production systems—on livestock farming systems and both livestock farming and cropping systems, respectively—through appropriate changes in farmland cover (i.e. changes in proportions of crops). We chose these scenarios as a result from an analysis of documents submitted in support of the European Commission proposals for the CAP 2013–2020 [58] and of case study documents [59, 60]. They correspond to different combinations of market trends and agricultural policies, not explicitly described in this paper. Two of them (*‘biofuel’* and *‘extensification’*) were done at two levels of implementation: national and regional. A national level means a spatially homogeneous policy implementation in all agricultural regions, regardless the type of their agro-ecosystem. Conversely, a regional level of implementation leads to spatially heterogeneous policies in the SAR, often ending in a specialization of agricultural territories. To account for these patterns, we thus developed six scenarios of farmland cover changes to further predict the potential impact of the evolution of agriculture on farmland birds:

- A *‘status quo’* scenario, in which the percentage of grasslands continues to decrease in cover nationally, especially permanent grasslands, due to agricultural abandonment, overall intensification, and plowing up of pastures within farms. The dominance of cereals persists and the phenomenon of “grain-fed livestock” noted in the last 20 years, continues to increase at the expense of grass and other forage crops.
- A *‘national biofuel’* scenario, which promotes the development of crops used for the production of biofuels based on a national policy implementation. In this scenario, we applied a national pattern of intensification typically associated with the development of bioenergy. This corresponds to an increase of cereal—oleaginous—proteaginous crops (COP) in most SAR, often at the expense of grasslands and forage crops.
- A *‘regional biofuel’* scenario, with similar objectives and changes as in the preceding but based on a regional policy implementation. In this scenario, the increase in COP areas within arable areas (including mixed-farming areas) is balanced by an extensification in livestock areas mainly driven by the implementation of an extensive management of grasslands.
- A *‘livestock extensification’* scenario, which promotes the extensification in livestock and mixed-farming areas, mainly by increasing grasslands and reducing forage maize, and status quo in arable areas (i.e. mimicking current cropping patterns).
- A *‘national extensification’* scenario, which promotes the overall extensification in all French agricultural regions following a same national political framework. It first imposes a fixed minimum proportion of grasslands throughout the country. This increase in grasslands is balanced according to agricultural characteristics of each agro-ecosystem. Indeed, in livestock areas and mixed-farming areas it mimics a change in the implementation of agri-environmental schemes related to the development of sustainable practices such as an increase in pasture cover and a more extensive management of grasslands, accompanied by a decrease of forage crops (including maize). In arable areas, it corresponds to an overall decrease of COP to ensure a minimum of 5% of permanent grasslands, and also to a better balance of rotations with an introduction of proteaginous or “new” crops.
- A *‘regional extensification’* scenario: extensification with a regional level of policy implementation. In arable and mixed-farming areas, it corresponds to an extensification of agricultural practices, resulting in an increase of area proportions dedicated to arable crops at the expense of grasslands. This is accompanied by a better balance of crop rotations and diversification of cropping systems (the primary crop should not exceed 45% of the agricultural area). The reduction of grasslands in arable and mixed-farming areas is partly offset by an extensification in grassland areas (i.e. livestock areas), corresponding to an increase of permanent grasslands surfaces at the expense of all other crops.

### Statistical methods

We evaluated the response of bird populations to the different farmland cover scenarios combined with changes in proportion of farmland habitats (land use change impact) and climatic suitability (climate change impact). With this aim, we tested several combinations of environmental changes. These were: (i) climate change only; (ii) changes in proportion of farmland habitats only; (iii) farmland cover scenarios only and (iv) farmland cover scenarios combined with only changes in proportion of farmland habitats (i.e. without climate change). All variables were included in these successive models, however all variables except those of interest (i.e., climate, proportion of farmland habitats, farmland cover or both farmland cover and proportion of farmland habitats) were kept at their current values. In order to focus on specific effects of agricultural changes on compare the different farmland cover scenarios, we further standardized the climate effect.

In a first step, we developed a calibration model, based on a habitat association model (M4 in Fig. 2), to estimate the variations in regional relative species abundances according to the proportions of farmland habitat and farmland cover (i.e. crops), and their climatic suitability in each SAR. To this purpose, we used generalized linear mixed models (GLMM) assuming a Poisson distribution of the data with a log link function. The abundance was considered as the dependent variable, whereas the climatic suitability, the proportion of farmland habitats and the nine crop proportions (permanent grassland, temporary grassland, cereals, grain maize, rapeseed, sunflower, corn fodder, proteaginous and forage crops) were defined as fixed effects. The number of farmland count points in the SAR was integrated as a covariate to account for the varying sampling effort (between SAR and between years within SAR). The fixed-effect structure is as follows:

$$Log(Abundance)\;\sim \;\alpha +\beta_{1}\times Climatic\;suitability\;+\beta_{2}\times Farmland\;habitats\;+\;\beta_{3}\times Number\;of\;Points\;+\beta_{x}\;\times Crops$$

Independently of the observation effort, counts were suspected not to be independent according to the SAR and the year they were carried out. Therefore, these two variables (‘SAR’ and ‘Year’) were specified as random effects in the model. There were signs of overdispersion for several species, so we added an observation-level random effect to the corresponding species models [61]. Fitting a log-normal Poisson model (with “observational level random effects") is one of the most common alternatives to deal with overdispersion [62] (see S2 Text for further details). Overdispersion was calculated following the methods in Harding & Hilbe [63] for each species data set. We first measured the model fit by assessing the ‘variance explained’ by the GLMM model for each species using the method from Nakagawa & Schielzeth [64] to estimate R² for mixed-effects models. We also used the predictions derived from the GLMM models, based on half of the regional data (training data), to assess the predictive power of the models when applied to the other half (test data). The predictive power of the models was determined by plotting the predicted relative abundance for test data (according to the model calibrated with the training data) of each species against the observed relative abundance of each species per SAR per year, and then fitting a regression line with an intercept of zero and a slope of 1 (i.e., y = x), from which an *R* ^*2*^ value was calculated. We did this for each of the 34 bird species.

The model calibration was performed using test data representing only half of the regional data gathered to explain the relative abundances of the other half of sampled regions. We then derived a prediction of the current regional abundance of each bird species as a baseline for assessing the effect of the different scenarios, from the estimates of the model outputs using full datasets. Indeed, once the estimates of the coefficients were obtained for each predictor, we calculated the predicted current (RAcurrent) and future (RAfuture) regional abundances (log scale) using respectively current and projected values of climatic suitability, proportions of farmland habitats and crop proportions, from the set of models described above. After back-transformation, we calculated changes in a species regional abundance RA as: [RAfuture—RAcurrent]/[(RAcurrent + RAfuture)/2] [65] (see details in S3 Text).

Finally, we examined differences between responses (mean changes in abundance) to farmland cover scenarios, and if they were related to species habitat specialization (farmland specialist vs. generalist). The latter was defined following Julliard et al. [48] who quantified specialization of a given species by calculating the variation in its density among various habitat classes (provided by BBS observers). We further explored potential relationship between farmland specialists’ responses and their main habitat of specialization: grassland, cropland and mixed grass/crop lands. Based on Julliard et al. (2006) methodology, main habitat of farmland specialists was determined with the calculation of a species specialization index for grasslands (SSIg). The SSIg was computed as a weighted mean of species abundance among four sub-habitats of the farmland habitat: unimproved grasslands, improved grasslands, mixed grass/crop lands and croplands, weighting coefficient of these sub-habitat being 4, 3, 2 and 1, respectively. Species with highest SSIg values (SSIg > 2.2) were considered as grassland birds, while species with lowest SSIg values (SSIg < 1.8) were considered as cropland birds; remaining intermediate species were considered as mixed habitat farmland species (see Table 1). We used one sample Student’s t-tests to check if mean changes in abundance in response to farmland cover scenario were significantly different from the null hypothesis (i.e. mu = 0). Because all SAR differ in area and number of count points, changes in regional abundance were weighted by the ratio of the area of corresponding SAR on the number of count points in all analyses in order to obtain comparable measures across SARs. Standard deviations associated with weighted mean changes were computed following Cochran’s 1977 definition [66]. We finally performed Tukey HSD (Honestly Significant Difference) tests, a single-step multiple comparison procedure, to check whether the mean changes in abundance, for each ‘farmland cover’ scenario and group of species with different SSI, were significantly different from each other. We performed all our statistical analyses using R [67] and used ‘nlme’ package for mixed models [68].

## Results

### Overall changes in bird populations in response to scenarios

**Fig 3:**
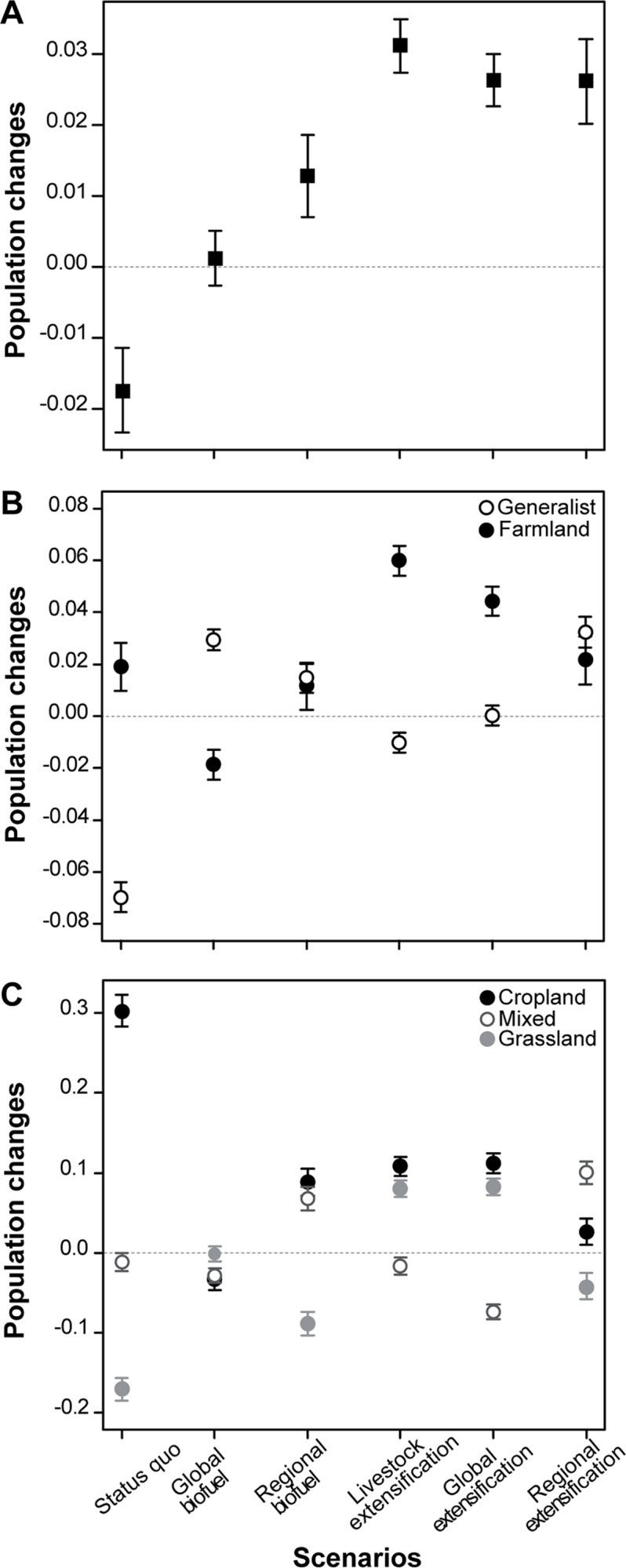
Predicted responses of bird populations to each farmland cover scenario. Mean (± SD) changes in regional abundance of A) all bird populations, B) farmland and generalist bird populations and C) grassland, cropland and mixed (grassland/cropland) bird populations according to each scenario of farmland cover changes (all combined to climate and land use change scenarios). Mean population changes correspond to the average of the changes in species regional abundance weighted by the ratio of the area of corresponding region (SAR) on the number of count points (see Methods). Mean population change under climate change alone constitutes the baseline reference and was fixed to 0. Significance of responses (from Student t-test) and significant differences between scenarios for each group of species (from Tukey HSD test) are given in S7–S10 Tables.

Most of the predictive models fitted well to the data (averaged *R²* across species was 61.5% ± 9.7). The model performed best for the Skylark (R² = 84%), while it had the lowest performance for the Grey Partridge (R² = 38%). Nevertheless, the correlations between the predicted and observed species abundances were significant for all species (results not shown). Our results show that the climatic scenario is expected to lead to more negative effects on bird abundances than under the land-use scenarios considered (see Table 2). However, the predicted mean changes in abundance of bird populations are lower when land use and farmland cover changes are added to climate changes than when climate change is considered solely (Table 2). Although the average response of bird abundance over all farmland cover scenarios (combined to land use and climate changes) may not be significantly different from the mean response to the climate change only scenario (Table 2), the Fig. 3a. illustrates that most of the farmland cover scenarios, except the ‘status quo’ and the ‘global biofuel’, can mitigate the impact of climate change on the overall bird populations (the zero-line on the graph). Changes in farmland area—a nationwide 4% decrease—also lead to negative mean changes in abundance of bird population, but less severe than climate. The mean changes in bird population sizes vary significantly according to the farmland cover scenario considered (Fig. 3a) and according to species (S6 Table). The *‘status quo’* scenario has a significant negative impact (*t* = -3.66, *p*-value <0.001), relative to all other scenarios that have positive or null (for the *‘global biofuel’* scenario) predicted impacts on bird populations. The mean changes in bird abundance are predicted to be the most positive with the three extensification scenarios (Fig. 3a; see S7 Table for significance), especially the *‘livestock extensification’* scenario (*t* = 10.25, *p*-value <0.001), but we find no significant difference between these different scenarios of agricultural extensification (S8 Table).

**Table 2:** Mean changes and associated standard deviation (SD) in regional abundance of all bird populations in response to changes in different environmental drivers: climate, land use (i.e. proportion of farmland habitats) and farmland cover scenarios.

Environmental changes	Mean	SD
Climate	-0.255	0.008
Land Use	-0.036	0.001
Farmland cover	0.037	0.002
Land Use + Farmland cover	0.005	0.002
Climate + Land Use + Farmland cover	-0.242	0.004

‘Environmental changes’ indicates variables allowed to change (current vs. future predictions) depending on each case scenario (the other variables are considered to remain the same in current vs. future predictions). All variables were included in all models though. The mean estimates associated with ‘Farmland cover’ correspond to the average response of bird abundance over all farmland cover scenarios.

### Relationship between species response and their habitat specialization

There is a significant effect of habitat specialization on the way species responded to the different farmland cover scenarios. Predicted mean changes in abundance of farmland and generalist bird populations differ significantly according to farmland cover scenarios (Table 3 and S7 Table). The *‘status quo’* scenario negatively impacts generalist bird populations, but has a positive effect on farmland bird populations (Fig. 3b and S7 Table). This is particularly apparent for cropland species which exhibit a strong positive response to this *‘status quo’* scenario, as opposed to grassland species which are negatively impacted (Fig. 3c, see also Table 3 and S9 Table). The *‘livestock extensification’* and *‘global extensification’* scenarios have significantly more positive impact on farmland species (respectively: mean ± SE = 0.060 ± 0.005 and 0.044 ± 0.005, see Fig. 3b). In particular, grassland and cropland species respond positively to these two scenarios, whereas mixed species respond negatively (Fig. 3c and S10 Table for significance). Generalist species respond positively to the *‘global biofuel’* scenario, whereas farmland species do not (Table 3). There is no significant difference between the responses of farmland specialists and generalists to the *‘regional biofuel’* and *‘regional extensification’* scenarios (Table 3). Within farmland specialists, however, these two scenarios lead to increasing mixed and cropland bird populations, as opposed to grassland species which exhibit decreasing abundances (Fig. 3c; Table 3).

**Table 3:** Differences between mean changes in populations of farmland and generalist bird species, and between mean changes in populations of farmland specialists (grassland, cropland and mixed species) in response to each farmland cover scenarios.

	*Farmland sp vs*. *Generalist sp*				*Grassland vs*. *Cropland*				*Grassland vs*. *Mixed*				*Cropland vs*. *Mixed*			
Scenarios	Diff	Lower	Upper	P-values	Diff	Lower	Upper	P-values	Diff	Lower	Upper	P-values	Diff	Lower	Upper	P-values
Status quo	0.088	0.062	0.115	**< 0.001**	-0.474	-0.519	-0.429	**< 0.001**	-0.16	-0.115	-0.205	**< 0.001**	0.315	0.363	0.266	**< 0.001**
Global Biofuel	-0.049	-0.075	-0.022	**< 0.001**	0.032	-0.013	0.077	0.612	0.027	0.072	-0.018	0.876	-0.005	0.043	-0.053	1.000
Regional Biofuel	-0.004	-0.030	0.023	1.000	-0.177	-0.222	-0.132	**< 0.001**	-0.157	-0.112	-0.202	**< 0.001**	0.021	0.069	-0.027	0.998
Livestock Extensification	0.070	0.043	0.096	**< 0.001**	-0.028	-0.073	0.017	0.832	0.097	0.142	0.052	**< 0.001**	0.125	0.173	0.077	**< 0.001**
Global Extensification	0.043	0.017	0.070	**< 0.001**	-0.030	-0.075	0.015	0.736	0.157	0.202	0.112	**< 0.001**	0.187	0.235	0.139	**< 0.001**
Regional Extensification	-0.011	-0.038	0.015	0.960	-0.068	-0.113	-0.023	**< 0.001**	-0.142	-0.097	-0.187	**< 0.001**	-0.074	-0.026	-0.122	**< 0.001**

Differences between mean changes in regional abundance of the different group of farmland specialists were calculated using Tukey HSD. Lower and upper values of the 95% confidence interval and adjusted p-values are also given.

### The effect of regionalization of public policies on bird populations

**Fig 4:**
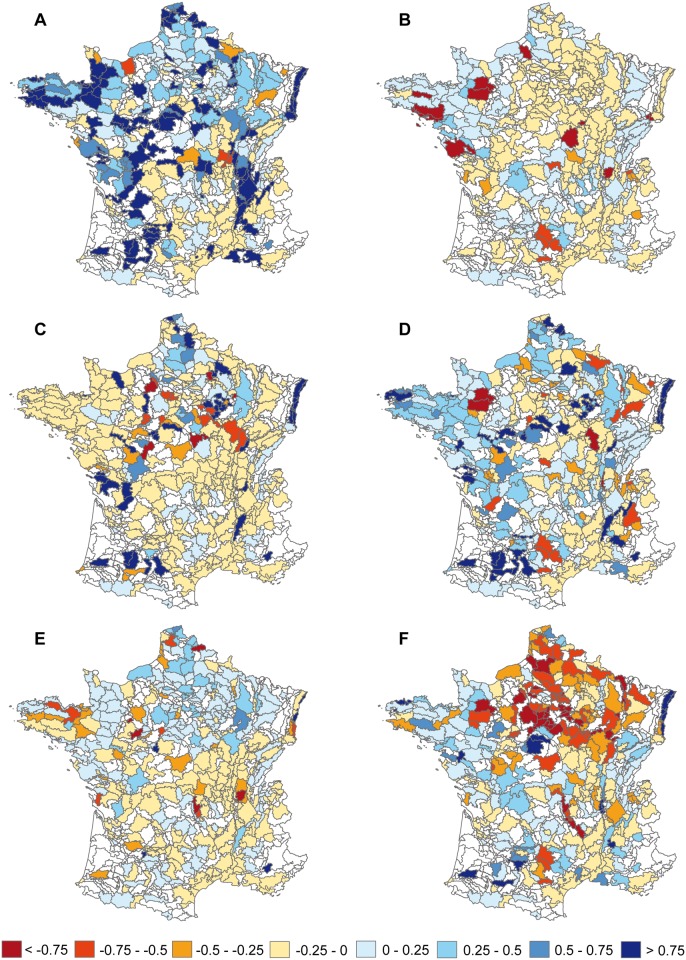
Predicted regional responses of Skylark *Alauda arvensis* to the different scenarios. Percent change in regional abundance of Skylark (one of the cropland specialist species) in response to the different land cover scenarios: A) *‘status quo’*, B) *‘livestock extensification’*, C) *‘global biofuel’*, D) *‘regional biofuel’*, E) *‘global extensification’* and F) *‘regional extensification’*, all combined to climate and land use change scenarios. The more red it is the more abundance decreases.

**Fig 5:**
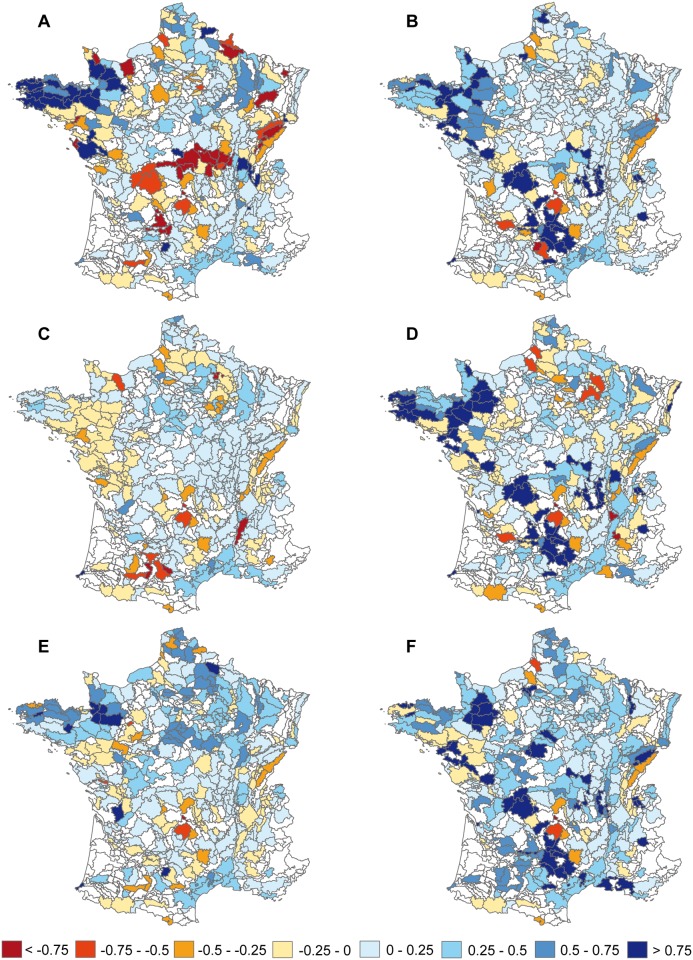
Predicted regional responses of Whinchat *Saxicola rubetra* to the different scenarios. Percent change in regional abundance of the Whinchat (one of the grassland specialist species) in response to the different land cover scenarios: A) *‘status quo’*, B) *‘livestock extensification’*, C) *‘global biofuel’*, D) *‘regional biofuel’*, E) *‘global extensification’* and F) *‘regional extensification’*, all combined to climate and land use change scenarios. The more red it is the more abundance decreases.

The *‘regional biofuel’* scenario has significantly more positive impact on farmland specialists than the *‘global biofuel’* scenario, whereas the *‘global extensification’* scenario has more positive impact than the *‘regional extensification’* scenario (Fig. 3b; S7 and S8 Tables). The situation is reversed for non-specialist species. Between global and regional scenarios, however, species exhibit various regional abundance changes that do not systematically correspond to the mean responses of all species (or group of species). For example, the skylark (one of the cropland specialist species) strongly exhibits contrasted changes in regional abundance in response to the *‘global extensification’* vs. *‘regional extensification’* scenarios, especially in arable areas of northern France (Fig. 4). For the whinchat *Saxicola rubetra* (one of the grassland specialist species) changes in regional abundance are predicted to be quite similar in response to both ‘*global’* and *‘regional’* extensification scenarios, in contrast to ‘*global’* and *‘regional’* biofuel scenarios (Fig. 5).

## Discussion

First, we showed that future climate changes, as predicted by the IPCC assessment reports [41], should have strong negative impacts on bird population sizes in farmland landscapes. This predicted negative impact of climate change on birds follows the trend of that observed for over the last decades around the world [69] and for European birds [21]. So, our results are in line with current knowledge on the role of climate in shaping bird populations [e.g. 23, 70]. Furthermore, our study is consistent with research on the coupled effect of climate change and land use [28, 30, 71]. We also found evidence that farmland use changes can mitigate the negative effect of climate change for some species or habitat guilds. This result support the hypothesis from Eglington and Pearce-Higgins [35] and Dormann et al. [33] that there is a potential for countering negative effects of climate change by adaptive landscape management. Our results also emphasize the needs to consider land use/cover when predicting large-scale changes in biodiversity [33, 34]. Nevertheless, in our study the climate effect significantly reduces abundance of some bird species in response to likely changes in agriculture. At broad spatial scales it is often possible to identify factors that limit maximum abundance without completely explaining or fully determining abundance in all places [72]. Environmental suitability may predict the upper limit of abundance better than mean abundance, making it particularly difficult to accurately predict local abundance under the influence of climate [72, 73].

Our results predict that bird populations will continue to decline strongly if current observed trends in agriculture persist during the coming decades. This is consistent with other studies on past and potential future effects of agricultural intensification on bird species [9]. In our study, this effect is particularly strong for generalist species that live in farmland, while all specialist species but grassland species seem not to be impacted. This result seems partly conflicting with studies relating agricultural intensification to farmland bird declines [74, e.g. 75, 76], but is consistent regarding trends in French populations in recent years. During the last decade, the rate of population declines of farmland specialists in France seems to have decreased [47]. Moreover, more than reflecting an intensification of practices—via higher input or mechanization—the *‘status quo’* scenario reflects a unification of farmland cover in arable areas and a drastic reduction of permanent grassland throughout the country. Such a scenario is indeed more favorable to open-habitat species described in this study as cropland species such as skylark or corn bunting *Emberiza calandra*, which in our results show a strong positive response to this scenario. This effect on open-habitat species pulls upwards the mean changes in abundance of farmland bird population. Meanwhile, it hides the strong negative effects of this scenario on the grassland species. As such, a *‘status quo’* scenario may lead to small and species-poor communities by promoting only few very specialized species among farmland birds.

Similarly, we found few positive effects of *‘biofuel’* scenarios on bird populations, and negative effects on farmland birds with the *‘global biofuel’* scenario. In the latter, the homogenization of crops for rapeseed oil in arable areas and the decrease of grasslands in all agricultural areas are detrimental to farmland birds throughout the study area. As a result, this scenario is predicted to increase the biotic homogenization already observed in farmland by favoring generalist species [77]. In contrast to the global scenario, the *‘regional biofuel’* scenario had a small positive impact on farmland and generalist birds. The combination of a regional extensification in livestock areas (i.e. increasing grasslands) and changes of mixed areas into more crop specialized areas seems to be more favorable to farmland birds. This is especially the case for crop specialists (e.g. skylark) and mixed cropland/grassland specialists (e.g. linnet). Although the *‘regional biofuel’* scenario partially compensates grassland decrease in arable and mixed-farming areas by increasing their habitats in livestock areas, the strong negative impact of this scenario on grassland species indicates that the regional extensification of grasslands to counterbalance biofuel development and crop intensification may be not effective. As a result, although less favorable for generalists, this scenario may not promote a diversified community of specialists as it favors only those specialized in open cropped habitats.

Extensification scenarios seem to be able to reverse current negative bird population trends in French farmland. Especially, two scenarios—*‘regional extensification’* and *‘livestock extensification’*—have the most positive response by bird abundance. This was mostly driven by a strong effect on farmland specialists. These two scenarios consider an increase of permanent and/or temporary grassland surfaces and an extensive management of these grasslands or crops (e.g. by diversifying rotations) in livestock and mixed-farming areas. These positive results are consistent with several studies that highlight a positive relationship between bird species abundances and semi-natural grasslands [e.g. 78, 79] and with recommendations that have been made on low-intensive management of grasslands to benefit birds [e.g. 80, 81]. Our results show that the combination of extensification in livestock areas and diversification of crops in arable areas as developed in the *‘global extensification’* scenario have a more positive impact on birds, especially on farmland specialists, than the combination with biofuel crops (tending towards monocultures). This supports conclusions of previous studies that find landscape heterogeneity generally benefits biodiversity [82–85], but it could be sometimes at the expense of specialist species or of species of conservation concern [SPECs, 86]. Indeed, here we found that farmland species whose primary habitat is mixed production systems (cropland and grasslands) are negatively impacted by the *‘global extensification’* scenario, as well as the *‘livestock extensification’* scenario, whereas they benefit more from the regional extensification. These species are the less specialized of the farmland specialists, and they appear to respond like the generalists to the three scenarios of extensification. Finally, in a *‘regional extensification’* scenario, agricultural practices such as diversification of crops or extensive management of grasslands seem to benefit both farmland and non-farmland specialists. However, if diversity-enhancing measures benefit non-farmland populations they do not favor some of the farmland specialist species which are more at risk [77].

The regionalization of agricultural trends leads to heterogeneous changes in farmland cover between SARs. In the case of *‘biofuel’* scenarios, the impact of biofuel crop cultivation on bird biodiversity depends on a combination of various effects at local or landscape scales [13, 87, 88], such as the choice of crops, geographical location, scale of implementation and the spatial distribution of the biofuel crops. This is also the case for extensification scenarios, in which the impact of contrasted variations in grassland extensification and crop diversification, either spatially or quantitatively, leads to contrasted responses of bird populations and does not benefit the same species. However, although a regional implementation provides more benefits to bird populations, the chosen crops largely determine the range of farmland specialists that are favored. Directly related to this regionalization policy, one important point is on mixed-farming systems (corresponding to mixed farming and diversification after livestock breeding; S2 Table). These systems represent a significant part of French farmland (30%). Given the results of the different extensification scenarios, it seems that agricultural extensification measures in these areas, such as increasing proportion of grasslands (permanent or temporary) and increasing diversity of crop rotation, reinforce the positive impact of extensification in livestock areas.

The positive effect of regionalization according to farmland cover scenarios varies between species. For instance, skylark generally responds more negatively to *‘regional extensification’* scenarios, especially in arable areas, than to the *‘global extensification’* scenario. These results can be explained by the preference of this species (or others such as yellowhammer) for low landscape diversity [89–91]. Conversely, regional scenarios, either *‘biofuel’* or *‘extensification’*, may favour grassland or semi open-species (e.g. whinchat) known to prefer diversified landscape.

By developing farmland cover scenarios that we combined with available scenarios of global environmental changes (i.e., climate and land use), we projected potential impacts of changes in crop patterns on farmland bird populations. We believe our approach is innovative since no comparable policy impact assessment that integrates both climate and land-use changes at these relatively fine spatial scales has yet to be applied to such a large set of bird species at a national scale. Nevertheless, one should note that these scenarios cannot be interpreted as predictions of future land uses, but rather as sets of coherent and internally consistent simulations based on plausible but necessarily simplified assumptions of how future farms may develop. These kinds of scenarios should always be interpreted in comparison with the reference scenario (here, the *‘status quo’* scenario). In addition, since we did not integrate potential effects of future climate changes on crop phenology, crop structure and yields, our results on the ability of land use to balance climatic effects should be interpreted cautiously. Our model relied on simple habitat associations and our projections are based on correlative analysis, which assumes stationarity. As in niche models, we assumed a high degree of niche conservatism between species occurrence-climate and abundance-land use relationships. Thus, it ignores consequences of species adaptations to changing climate and land use [33]. The uncertainty attached to violations of these assumptions cannot be estimated and is hence inevitably ignored [92]. Besides, some temporal mismatches between land use and bird data inputs may bias our forecasting results. We believe, however, that land use changes at such large spatial scale happen slower than land cover data. We emphasize the need to develop more land use scenarios at various spatiotemporal scales based on data that can match with the existing biological data sets, in order to make more accurate predictions from this type of scenario-based studies. It is also important to note that the value of our estimates is mainly heuristic, but our results therefore illustrate some possible consequences for biodiversity as a result of adopting various agricultural development pathways in the future.

Nevertheless, there are important conclusions that can be drawn from this scenario-based study. Our results confirm the importance of modeling land cover together with climate change when predicting future biodiversity changes in response to these key drivers, also because in some scenarios land use changes compensated partly the negative impacts predicted from climate change alone. The contrasted responses of bird populations to the different farmland cover scenarios, combined with land use changes, show that agricultural changes can be important drivers of bird diversity. A scenario of more intensive farmland is likely to contribute to further declines of breeding bird abundances in farmland habitats. Additional management efforts counteracting the decrease in landscape heterogeneity are thus needed. Increase or rehabilitation of grassland areas regionally or throughout the country, extensive management of both grasslands and crops areas, and increasing heterogeneity in arable areas might benefit bird populations, or even reverse the current trends, especially those of farmland birds. Such positive impacts justify the introduction or reinforcement of these extensive practices in the common agricultural policy. The extensification of farmland cover seems more appropriate than following with the current agricultural trends or the development of bioenergy crops. However, the effects of extensification on farmland birds differ markedly depending on region and landscape structure, and also depending on species habitat requirements. Consequently, conservation measures need to account for the regional differences in farming practices and landscape structure. These results can contribute to evaluations of public decisions for support in agriculture and biodiversity management. For this purpose, this study might be complemented by other approaches incorporating economic drivers such as agricultural prices and subsidies, which may affect the returns of different land use/cover patterns [see 93, 94]. Finally, Concepción [95] has recently demonstrated that responses of organisms to agricultural change are strongly non-linear and dependent on landscape configuration. To better predict species responses, we suggest that further development of such farmland cover scenarios should integrate some landscape metrics which have strong effects on the abundance of bird populations, such as metrics of fragmentation (number of patches and edge density) but also topography [34].

## Supporting Information

S1 Fig Proportion of farmland habitats within each small agricultural region (SAR). (A) Current and (B) future distribution of farmland habitats in France. These values were derived from the IMAGE 2.4 model developed at a 0.5° resolution grid and were downscaled at the scale of each SAR. Regional proportion of farmland habitats were calculated as the percentage of land area covered by: herbaceous or cultivated pasture, cultivated and managed areas, and mosaic cropland/natural vegetation. Future proportion of farmland habitats (for 2050) were obtained by averaging projected proportions from the three SRES scenarios, A1B, A2, and B1 of the IMAGE 2.4 model.
(DOCX)

S1 Table Mean values of the current and future species climatic suitability. The mean AUC (area under the relative operating characteristic curve) of the cross-validation is also given. See Methods section 2.2 for details on how those values were obtained.
(DOCX)

S2 Table Changes in main agroecosystem in each farmland cover scenario. Blanks mean that there is no change of agroecosystem, but do not mean any change in crop/grassland proportions.
(DOCX)

S3 Table Basic constraints linked to main agroecosystem, adapted from AND International (2008). (DOCX)

S4 Table No additional mu constraints were set for the ‘Global Extensification’ scenario, in order to meet the constraints linked to main agroecosystem and find an optimal solution to the linear program. (DOCX)

S5 Table National changes in crop proportions according to each farmland cover scenarios. (DOCX)

S6 Table Weighted mean and associated standard deviation of species population changes in response to the different farmland cover scenarios combined to both scenarios of climate and land use changes. (DOCX)

S7 Table Results of one sample Students t-test on mean changes on bird populations in response to each farmland cover scenario. Estimated mean, standard error, t- and p-values are given.
(DOCX)

S8 Table Results of Tukey HSD test given the difference (Diff) between mean changes in bird populations between scenarios for all species, farmland and generalist species. Lower and upper values of the 95% confidence interval and adjusted p-values are also given.
(DOCX)

S9 Table Results of one sample Students t-test on mean changes on farmland bird populations (i.e. grassland, cropland and mixed species) in response to each farmland cover scenario. Estimated mean, standard error, t- and p-values are given.
(DOCX)

S10 Table Results of Tukey HSD test given the difference (Diff) between mean changes in farmland bird populations between scenarios for grassland species, mixed and cropland species. Lower and upper values of the 95% confidence interval and adjusted p-values are also given.
(DOCX)

S1 Text Development of farmland cover scenarios. (DOCX)

S2 Text Supporting information on how to account overdispersion. (DOCX)

S3 Text Supporting information on calculations of species regional abundance. (DOCX)
