# Supplementary material for: Forecasting the Effects of Land Use Scenarios on Farmland Birds Reveal a Potential Mitigation of Climate Change Impacts
Source: PLoS One. 2015 Feb 20;10(2):e0117850. doi: 10.1371/journal.pone.0117850 (PMC4336325; doi:10.1371/journal.pone.0117850)

**Figure S1. Proportion of farmland habitats within each small agricultural region (SAR)**. (A) Current and (B) future distribution of farmland habitats in France. These values were derived from the IMAGE 2.4 model developed at a 0.5° resolution grid and were downscaled at the scale of each SAR. Regional proportion of farmland habitats were calculated as the percentage of land area covered by: herbaceous or cultivated pasture, cultivated and managed areas, and mosaic cropland/natural vegetation. Future proportion of farmland habitats (for 2050) were obtained by averaging projected proportions from the three SRES scenarios, A1B, A2, and B1 of the IMAGE 2.4 model.


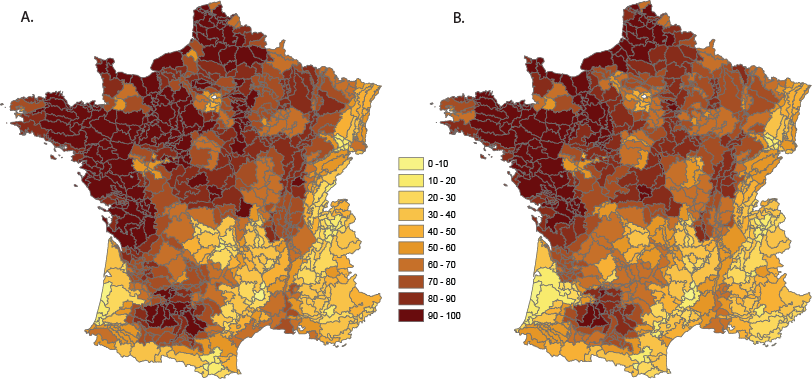

Supplement: S1 Fig — (A) Current and (B) future distribution of farmland habitats in France. These values were derived from the IMAGE 2.4 model developed at a 0.5° resolution grid and were downscaled at the scale of each SAR. Regional proportion of farmland habitats were calculated as the percentage of land area covered by: herbaceous or cultivated pasture, cultivated and managed areas, and mosaic cropland/natural vegetation. Future proportion of farmland habitats (for 2050) were obtained by averaging projected proportions from the three SRES scenarios, A1B, A2, and B1 of the IMAGE 2.4 model. (DOCX) [file pone.0117850.s001.docx]
