# Supplementary material for: Forecasting the Effects of Land Use Scenarios on Farmland Birds Reveal a Potential Mitigation of Climate Change Impacts
Source: PLoS One. 2015 Feb 20;10(2):e0117850. doi: 10.1371/journal.pone.0117850 (PMC4336325; doi:10.1371/journal.pone.0117850)
Supplement: S1 Table — The mean AUC (area under the relative operating characteristic curve) of the cross-validation is also given. See Methods section 2.2 for details on how those values were obtained. (DOCX) [file pone.0117850.s002.docx]

**Table S1**.Mean values of the current and future species climatic suitability. The mean AUC (area under the relative operating characteristic curve) of the cross-validation is also given. See Methods section 2.2 for details on how those values were obtained.

| **Species** | **Current climatic suitability** | **Future climatic suitability** | **AUC** |
| --- | --- | --- | --- |
| Grey Partridge *Perdix perdix* | 0.86029021 | 0.55688161 | 0.998603 |
| Yellow Wagtail *Motacilla flava* | 0.88001703 | 0.83609134 | 0.997101 |
| Corn Bunting *Emberiza calandra* | 0.93863444 | 0.92870028 | 0.998863 |
| Lapwing *Vanellus vanellus* | 0.75658985 | 0.35822746 | 0.998456 |
| Quail *Coturnix coturnix* | 0.93689069 | 0.95177192 | 0.998722 |
| Skylark *Alauda arvensis* | 0.9801765 | 0.95580075 | 0.999062 |
| Red-legged Partridge *Alectoris rufa* | 0.61662575 | 0.5515849 | 0.999048 |
| Linnet *Carduelis cannabina* | 0.98333719 | 0.94096715 | 0.999399 |
| Rook *Corvus frugilegus* | 0.64442428 | 0.28646812 | 0.997679 |
| Meadow Pipit *Anthus pratensis* | 0.60623092 | 0.13063837 | 0.998915 |
| Whitethroat *Sylvia communis* | 0.96375598 | 0.95833441 | 0.999188 |
| Kestrel *Falco tinnunculus* | 0.96304668 | 0.88945295 | 0.998527 |
| Yellowhammer *Emberiza citrinella* | 0.90527738 | 0.6119584 | 0.998743 |
| Stonechat S*axicola torquatus* | 0.9411831 | 0.94346234 | 0.998015 |
| Cirl Bunting *Emberiza cirlus* | 0.79143418 | 0.90045582 | 0.998447 |
| Buzzard *Buteo buteo* | 0.94962935 | 0.95337175 | 0.998873 |
| Whinchat *Saxicola rubetra* | 0.85765313 | 0.56613589 | 0.998969 |
| Hoopoe *Upupa epops* | 0.80087396 | 0.90110971 | 0.998095 |
| Red-backed Shrike *Lanius collurio* | 0.83409823 | 0.81527911 | 0.999043 |
| Wood Lark *Lullula arborea* | 0.83201247 | 0.88970023 | 0.998248 |
| Blackbird *Turdus merula* | 0.9846818 | 0.96942029 | 0.998793 |
| Blackcap *Sylvia atricapilla* | 0.97704388 | 0.91124142 | 0.999021 |
| Blue Tit *Cyanistes caeruleus* | 0.96222487 | 0.94602938 | 0.999055 |
| Carrion Crow *Corvus corone* | 0.9112975 | 0.63226483 | 0.998357 |
| Chaffinch *Fringilla coelebs* | 0.99166604 | 0.99291856 | 0.999376 |
| Cuckoo *Cuculus canorus* | 0.99085219 | 0.96566668 | 0.99967 |
| Dunnock *Prunella modularis* | 0.93066959 | 0.71145143 | 0.998716 |
| Golden Oriole *Oriolus oriolus* | 0.88688403 | 0.92351608 | 0.998981 |
| Great Tit *Parus major* | 0.97781852 | 0.96032098 | 0.999197 |
| Green Woodpecker *Picus viridis* | 0.96835794 | 0.94251694 | 0.998603 |
| Jay *Garrulus glandarius* | 0.98260223 | 0.97056424 | 0.99889 |
| Melodious Warbler *Hippolais polyglotta* | 0.7824316 | 0.78437285 | 0.999042 |
| Nightingale *Luscinia megarhynchos* | 0.92237876 | 0.96122543 | 0.998347 |
| Wood Pigeon *Columba palumbus* | 0.9570846 | 0.89009385 | 0.997971 |
