# Supplementary material for: Forecasting the Effects of Land Use Scenarios on Farmland Birds Reveal a Potential Mitigation of Climate Change Impacts
Source: PLoS One. 2015 Feb 20;10(2):e0117850. doi: 10.1371/journal.pone.0117850 (PMC4336325; doi:10.1371/journal.pone.0117850)
Supplement: S2 Table — Blanks mean that there is no change of agroecosystem, but do not mean any change in crop/grassland proportions. (DOCX) [file pone.0117850.s003.docx]

**Table S2**. Changes in main agroecosystem in each farmland cover scenario. (Blanks mean that there is no change of agroecosystem, but do not mean any change in crop/grassland proportions).

Abbreviations: LF, Livestock Farming; MLF, Mountain Livestock Farming; LLF, Lowland Livestock Farming; AC, Arable Crops; Diversification, Diversification after livestock farming. “rapeseed”, “rotations” (i.e. diversified rotations) and “specialization” specify main trends on Arable Crops
