# Supplementary material for: Forecasting the Effects of Land Use Scenarios on Farmland Birds Reveal a Potential Mitigation of Climate Change Impacts
Source: PLoS One. 2015 Feb 20;10(2):e0117850. doi: 10.1371/journal.pone.0117850 (PMC4336325; doi:10.1371/journal.pone.0117850)
Supplement: S3 Table — (DOCX) [file pone.0117850.s004.docx]

**Table S3**. Basic constraints linked to main agroecosystem, adapted from AND International (2008)

Abbreviations: AC, Arable Crops; ACA, Arable Crops Area; LF, Livestock farming; Diversification, Diversification after livestock farming; Mixed, Mixed farming; MFA, Main Forage Area; PGA, Permanent Grassland Area; UAA, Utilized Agricultural Area.
