# Supplementary material for: Forecasting the Effects of Land Use Scenarios on Farmland Birds Reveal a Potential Mitigation of Climate Change Impacts
Source: PLoS One. 2015 Feb 20;10(2):e0117850. doi: 10.1371/journal.pone.0117850 (PMC4336325; doi:10.1371/journal.pone.0117850)
Supplement: S4 Table — (DOCX) [file pone.0117850.s005.docx]

**Table S4**. Supplementary « mu constraints » linked to agroecosystems changes. No additional mu constraints were set for the ‘Global Extensification’ scenario, in order to meet the constraints linked to main agroecosystem and find an optimal solution to the linear program.

Abbreviations: LF, Livestock Farming; MLF, Mountain Livestock Farming; LLF, Lowland Livestock Farming; AC, Arable Crops; Diversification, Diversification after livestock farming. “rapeseed”, “rotations” (i.e. diversified rotations) and “specialization” specify main trends on Arable Crops
