# Supplementary material for: Forecasting the Effects of Land Use Scenarios on Farmland Birds Reveal a Potential Mitigation of Climate Change Impacts
Source: PLoS One. 2015 Feb 20;10(2):e0117850. doi: 10.1371/journal.pone.0117850 (PMC4336325; doi:10.1371/journal.pone.0117850)
Supplement: S6 Table — (DOCX) [file pone.0117850.s007.docx]

**Table S6.** Weighted mean and associated standard deviation of species population changes in response to the different farmland cover scenarios combined to both scenarios of climate and land use changes.
