# Supplementary material for: Forecasting the Effects of Land Use Scenarios on Farmland Birds Reveal a Potential Mitigation of Climate Change Impacts
Source: PLoS One. 2015 Feb 20;10(2):e0117850. doi: 10.1371/journal.pone.0117850 (PMC4336325; doi:10.1371/journal.pone.0117850)
Supplement: S7 Table — Estimated mean, standard error, t- and p-values are given. (DOCX) [file pone.0117850.s008.docx]

**Table S7**. Results of one sample Students t-test on mean changes on bird populations in response to each farmland cover scenario. Estimated mean, standard error, t- and p-values are given.
