# Supplementary material for: Forecasting the Effects of Land Use Scenarios on Farmland Birds Reveal a Potential Mitigation of Climate Change Impacts
Source: PLoS One. 2015 Feb 20;10(2):e0117850. doi: 10.1371/journal.pone.0117850 (PMC4336325; doi:10.1371/journal.pone.0117850)
Supplement: S8 Table — Lower and upper values of the 95% confidence interval and adjusted p-values are also given. (DOCX) [file pone.0117850.s009.docx]

**Table S8**. Results of Tukey HSD test given the difference (Diff) between mean changes in bird populations between scenarios for all species, farmland and generalist species. Lower and upper values of the 95% confidence interval and adjusted p-values are also given.
