# Supplementary material for: Forecasting the Effects of Land Use Scenarios on Farmland Birds Reveal a Potential Mitigation of Climate Change Impacts
Source: PLoS One. 2015 Feb 20;10(2):e0117850. doi: 10.1371/journal.pone.0117850 (PMC4336325; doi:10.1371/journal.pone.0117850)
Supplement: S10 Table — Lower and upper values of the 95% confidence interval and adjusted p-values are also given. (DOCX) [file pone.0117850.s011.docx]

**Table S10**. Results of Tukey HSD test given the difference (Diff) between mean changes in farmland bird populations between scenarios for grassland species, mixed and cropland species. Lower and upper values of the 95% confidence interval and adjusted p-values are also given.
