# Supplementary material for: Forecasting the Effects of Land Use Scenarios on Farmland Birds Reveal a Potential Mitigation of Climate Change Impacts
Source: PLoS One. 2015 Feb 20;10(2):e0117850. doi: 10.1371/journal.pone.0117850 (PMC4336325; doi:10.1371/journal.pone.0117850)
Supplement: S1 Text — (DOCX) [file pone.0117850.s012.docx]

**Text S1. Development of farmland cover scenarios.**

*This section gives information about the development of the farmland cover scenarios that has not been detailed in the manuscript.*

To be coherent, development of future agricultural scenarios must account for the diversity and dynamics of French agriculture. These dynamics include both production systems (specialization or diversification) and crop management (increase or decrease in intensity of practices). They result in various environmental pressures which are more or less specific depending on regional contexts. Each scenario represents a potential outcome of French agriculture according to realistic assumptions on future political decisions and changes in livestock farming and cropping patterns. Farmland cover scenarios are based on changes in the use of the Utilized Agricultural Area (UAA) in each small agricultural region (SAR), i.e. the variations of cropping patterns (i.e. crop/grassland proportions) between 2000 and 2050, respectively the base year for the agricultural data and the time horizon considered. For the development of scenarios, the SARs classified as " Livestock Farming" and "Semi-extensive Livestock Farming" were differentiated into two categories: Mountain Livestock Farming and Lowland Livestock Farming areas, by overlapping the map of the current main agroecosystems (see Figure 1.a.) with the map of the Less Favored Areas defined as "Mountain/hill Areas" (<http://ec.europa.eu/agriculture/rurdev/lfa/>). Farmers in LFAs are supported by payments from the EU Rural Development Regulations, with the aim to “contribute, through continued use of agricultural land, to maintaining the countryside as well as to maintaining and promoting sustainable farming systems”. In addition, the SARs whose main was defined as ‘Arable Crops dominated by maize’ were combined with the ‘Arable Crops specialization’ agroecosystems. In a first step, we defined changes in main agroecosystems according to each scenario, within the geographic conText Snd likely temporal dynamics of the different agroecosystems of each SAR (e.g. differences between mountain and lowland agroecosystems; see Table S2 and Figure 1). As an example, in a scenario of agricultural intensification (e.g. *‘status quo’* scenario), a SAR with currently extensive livestock farming could not change into a crop area, but might change into a more intensive livestock area. In a second step, we estimated the variations in the proportions of crops within each SAR linked to the changes of the main agroecosystem within each SAR, according to each scenario. We considered the following nine crops: permanent grassland, temporary grassland, cereals, grain maize, rapeseed, sunflower, forage maize, pulses and fodder crops. Changes in crop proportions were calculated using an optimization method taking various constraints into account (see Table S2 for the basic constraints linked to agroecosystems). The aim was to minimize the rate of change in farmland cover in each SAR, according to the range of possibilities in each agroecosystem (based on segmentation criteria of the agroecosystems [1]) that we defined as another set of constraints (see Table S3). Optimization calculations were performed with the GNU Linear Programming Kit (GLPK) and the associated command-line solver GLPSOL (<http://www.gnu.org/software/glpk/>; <http://en.wikibooks.org/wiki/GLPK>). The objective function was defined as follows:

$$\min\sum_{i,j} \left| \mathcal{x}_{ij}-p_{ij} \right|$$

where $p_{ij}$ and $\mathcal{x}_{ij}$ correspond respectively to the current and future proportions, in each SAR, of crop *i*, in the agroecosystem *j*. However, the problem was not linear, so we transformed as follows:

$$\min\sum_{i,j} \alpha_{ij}$$

subject to the following linear constraints:

$$\mathcal{x}_{ij}-p_{ij}\leq\alpha_{ij}$$

$$-\mathcal{x}_{ij}+p_{ij}\leq\alpha_{ij}$$

In addition to the set of constraints related to the characteristics of agricultural systems, mentioned above, two other sets of constraints were applied to solve the linear program. A first set of constraints has been defined in terms of changes in agricultural dynamics: when in a SAR, an agroecosystem *j_1_* (current) evolves into an agroecosystem *j_2_* (future) (see Table S2), the average future proportion of each crop in *j_1_* tends towards the average current proportion of the corresponding crop in *j_2_*. However, depending on the evolution of the agro- ecosystem this constraint has not been applied to all 9 rotations (Table S4), in order to meet the constraints linked to main agroecosystem and find an optimal solution to the linear program. Average proportions of each crop were calculated above all SAR of the same agroecosystem. A second set of constraints has been defined, based both on the characteristics of use of the UAA in each agroecosystem and on the objectives of cropping patterns specific to the different public policies implemented in each scenario (we do not provide the table with those constraints as it is hard to understand for a non-specialized reader). An example of constraint related to agroecosystem characteristics is the proportion of rapeseed, sunflower and proteaginous which cannot exceed respectively 40, 30 and 15% in the arable land surface. A last constraint was applied to all SAR: the sum of the surfaces in each SAR should be less than the agricultural land available. With regard to crop proportions, this was evidenced by the following constraint: $\sum_{i,j} (\mathcal{x}_{ij})\leq100$. The six farmland cover scenarios resulting from this method are summarized in the manuscript. The Table S5 illustrates the national changes in crop proportions according to each farmland cover scenarios.

1. AND International 2008. Evaluation ex-post du Plan de Développement Rural National 2000-2006; soutiens à l'agro-environnement. Ministère de l'Agriculture et de la Pêche.
