# Supplementary material for: Forecasting the Effects of Land Use Scenarios on Farmland Birds Reveal a Potential Mitigation of Climate Change Impacts
Source: PLoS One. 2015 Feb 20;10(2):e0117850. doi: 10.1371/journal.pone.0117850 (PMC4336325; doi:10.1371/journal.pone.0117850)
Supplement: S2 Text — (DOCX) [file pone.0117850.s013.docx]

**Text S2. Supplementary information on how to account overdispersion**

There is several modelling options to deal with overdispersion in count data with mixed models [1, 2]: (a) Poisson-lognormal models, including an “individual-level random effect”, as described by Elston et al. [3], (b) models with negative binomial distribution, (c) zero-inflated models, when there is an important substantial proportion of zero in datasets. Poisson-lognormal models and negative binomial models are qualitatively similar. The quasilikelihood estimation “traditionally” used to deal with overdispersion was deemed unreliable in lme4, and is no longer available. Part of the problem was questionable numerical results in some cases; the other problem was that Douglas Bates (lme4 R package) felt that he did not have a sufficiently good understanding of the theoretical framework that would explain what the algorithm was actually estimating in this case (see <http://glmm.wikidot.com/>) .

In our bird data set, some species had a high proportion of zero counts. However, we used Poisson-lognormal models to deal with overdispersion in our study. First, dealing with more than two random effects (as this in the case in our mixed models) in zero-inflated models implies too many hypothesis, and currently developed packages do not allow to simply model and understand the outputs as it is possible with GLMM. Besides, the addition of a random observation-level effect in a Poisson model is fluently used, especially as it said above it is similar to negative binomial models. It can easily be found in other studies with mixed models and count data, as well as in the useful forum R-sig-mixed-models (there are a lot of posts from Douglas Bates and Jarrod Hadfield about this topic). Agresti [4] also discusses this (section 13.5) in his book.

1. Rigby RA, Stasinopoulos DM and Akantziliotou C (2008) A framework for modelling overdispersed count data, including the Poisson-shifted generalized inverse Gaussian distribution. Computational Statistics & Data Analysis 53: 381-393.

2. Bolker BM, Brooks ME, Clark CJ, Geange SW, Poulsen JR, et al. Generalized linear mixed models: a practical guide for ecology and evolution. Trends in Ecology & Evolution 24: 127-135.

3. Elston, D. A. et al. 2001. Analysis of aggregation, a worked example: numbers of ticks on red grouse chicks. — Parasitology 122: 563-569.

4. Agresti, A. 2007. Categorical Data Analysis. 3rd ed. Hoboken, NJ: Wiley.
