# Supplementary material for: Forecasting the Effects of Land Use Scenarios on Farmland Birds Reveal a Potential Mitigation of Climate Change Impacts
Source: PLoS One. 2015 Feb 20;10(2):e0117850. doi: 10.1371/journal.pone.0117850 (PMC4336325; doi:10.1371/journal.pone.0117850)
Supplement: S3 Text — (DOCX) [file pone.0117850.s014.docx]

**Text S3. Supplementary information on calculations of species regional abundance.**

For each SAR, species regional abundance RA was calculated following the methods of Lemoine *et al.* (2007). In contrast to the usual calculations of percent change, we used the mean of regional current and future abundance as the denominator, not the regional current abundance. Using the regional current abundance as denominator would produce asymmetrical increases and declines; the maximum decline a species could achieve was −1 and the maximum increase was much higher than +1. The use of the mean of the regional current and future abundance as denominator has the advantage of producing symmetrical increases and declines; the maximum decline in the case of extinction was −2, and maximum increase in the case of colonization was +2.
